# Male Majority, Female Majority, or Gender Diversity in Organizations: How Do Proportions Affect Gender Stereotyping and Women Leaders’ Well-Being?

**DOI:** 10.3389/fpsyg.2019.01037

**Published:** 2019-05-09

**Authors:** Melanie C. Steffens, Maria Angels Viladot, Carolin Scheifele

**Affiliations:** ^1^Department of Social, Environmental, and Economic Psychology, Faculty of Psychology, University of Koblenz and Landau, Landau, Germany; ^2^Estudis de Psicologia, Universitat Oberta de Catalunya, Barcelona, Spain

**Keywords:** gender equality, gender diversity, stereotyped attitudes, leadership, role conflicts, organizational politics

## Abstract

Whereas popular wisdom often centers on character differences between women and men when explaining work-related behavior, Kanter (1977) predicted that the proportion of women and men present in organizations is the crucial factor: With unequal proportions, women (similar to other minority persons) are singled out as “tokens” and gender becomes salient, which has been theorized to have a range of negative consequences. In contrast, if proportions of women and men are similar (i.e., in the presence of gender diversity), gender is not salient, and the work environment becomes much more positive for women. These considerations imply that not only a male majority, but also a female majority at work has negative consequences, because gender becomes salient in both cases. However, empirical research on work environments with female majorities at the top of organizations is scarce. The present study tested the perception of a range of negative consequences, including work-related well-being, among women in leadership positions in Spain who reported a male majority, a female majority, or similar proportions of both genders at the top level of their organization. The online convenience sample consisted of a total of *N* = 649 women leaders. In addition to work-related well-being, we measured perceived work–family conflict and perceived feelings of guilt associated with work–family conflict, traditional gender stereotypes regarding warmth and competence, women-leadership stereotypes, negative work-related stereotypes of mothers, gender harassment, and stigma consciousness. Almost all of our findings support Kanter’s theorizing that equal proportions of both genders go along with more positive perceptions as compared to a male majority. However, a female majority went along with as negative perceptions as a male majority regarding several of the outcome variables, but was associated with the most positive perceptions for other outcomes. We discuss implications and possible reasons for the latter mixed findings.

## Introduction

In the work sphere, gender diversity has been especially scarce at the leadership level. Despite progress, only 24 companies of the Fortune Global 500 (i.e., 4.8%) have female CEOs. In Europe, the share of women in Executive Committees of the top 100 companies remains at 15% (Catalyst, 2018). Past research has obtained mixed results regarding gender diversity (or the lack thereof) and the benefits of heterogeneous versus homogeneous work teams (Bowers et al., 2000; Joshi and Roh, 2009; Shore et al., 2009). The objective of the present study was to examine the effects of different gender compositions at the top-level of organizations on a whole range of potentially important outcome variables. Drawing on Kanter’s (1977) seminal paper on gender proportions in organizations, we set out to examine the consequences of balanced gender compositions as well as male or female majorities for female leaders. As work has a significant impact on women’s well-being (Campione, 2008), we included a measure of work-related well-being besides outcomes related to stereotyping and sexism. We introduce each of the concepts (written in italics) below after providing some background on women’s situation in Spain, where the present study was carried out.

Spain is a country that has faced rapid changes following the end of the Franco regime in 1975 and economic challenges due to the global financial crisis (Hernandez Bark et al., 2014). Staying abreast of these changes, the Spanish prime minister has recently introduced a government with a female majority (Morrin, 2018). Moreover, the percentage of women in top positions in Spain is 27%, thus higher than the average in Europe (Catalyst, 2018). As compared to other southern-European countries, people in Spain live in more urban areas, consider religion less important, and hold more liberal gender-role attitudes (Kuyper et al., 2013). Nevertheless, Spanish women still spend an average of 4.5 h every day doing household duties, including childcare and elderly care (Instituto Nacional de Estadística [INE], 2016). A previous study with 145 women in leadership positions in Spain reported that they thought the main factors hindering gender diversity in leadership were related to organizational cultures: the long working hours expected of people in leadership positions, the competitive style, and little sensitivity for the need to balance work and family-related obligations (Chinchilla et al., 2003). Interestingly, almost half of the women (43%) believed the main factor that hindered their career was the lack of representation of women in leadership positions. Why could the proportion of women matter?

Gender is one of the social categories used most in work contexts (Eagly and Karau, 2002). It influences people’s reactions toward individuals and their interactions (e.g., Ito and Urland, 2003). Importantly, Kanter (1977) postulated that this process of social categorization depends on the gender composition in the environment. If women are in the minority, forming 15% of the work group or less (i.e., skewed proportions), they acquire token status. Tokens are often the pioneers who occupy positions in which historically disadvantaged groups were not present. According to Kanter, when women account for more than 15% and up to 35%, tilted proportions are given. From a share of 40% upward, balanced proportions or, in other words, gender diversity has been reached. Focusing on skewed proportions, Kanter proposed several perceptual outcomes: visibility, polarization, and assimilation. These perceptual outcomes suggest that token women are highly visible for majority members; the differences between women and men are exaggerated and polarized; and women’s features and behaviors are assimilated to stereotypes. As consequences, token women can experience performance pressure, heightened group boundaries, isolation, and role entrapment (Kanter, 1977).

Both the basic hypothesis that token status affects women more negatively than tilted or balanced proportions as well as the specific outcomes of token status have been tested. For example, women working in skewed or tilted settings with male majorities were less satisfied with their job and had higher intentions to resign (Burke and McKeen, 1996). In addition, gender-balanced organizations seem to trump male-dominated ones regarding equal evaluations of women’s and men’s success (Choi and Hon, 2002). However, support for Kanter’s hypothesis is inconclusive (Peccei and Lee, 2005; Stichman et al., 2010), and several confounding factors have been proposed (e.g., gender-atypicality of the occupation, gender status, or job prestige; Yoder, 1991, 1994, 2002; Hewstone et al., 2006).

Clarification is also needed for other gender compositions besides female token status. According to Kanter (1977), increasing numbers of women would lead to less negative outcomes for women because they have more opportunities to form coalitions, have more influence on the culture of the overall group, and are increasingly perceived as individuals. In contrast to this assumption, Yoder (1991) suggested that men would feel threatened by larger female proportions resulting in increased discrimination and harassment. Moreover, a consideration of the effects of female majorities in work groups in contrast to male majorities is scarce (but see Burke and McKeen, 1996; Hewstone et al., 2011). We assume that not only a male majority, but also a female majority at work has negative consequences, because gender becomes salient in both cases. Against this backdrop, the aim of the present study is to gain insights into the effects of different gender compositions at the top-level of organizations (male majorities, female majorities, and gender diversity) by examining various outcome variables described in the following.

As mentioned, token status can lead to perceptions of individual women being assimilated and interpreted in accordance with gender stereotypes. Women’s attributes and behaviors are thus increasingly overgeneralized and viewed through a lens of stereotypes (Kanter, 1977; Whittock, 2002). Hewstone et al. (2006, 2011) found support for these assimilation effects: Proportions with female underrepresentation, especially skewed ones, led to increased perceptions of women’s homogeneity by both men and women.

The content of gender stereotypes is often organized according to the basic stereotype dimensions, agency and communion (Bakan, 1966). Women are traditionally associated with communal traits such as being warm and caring, while men are associated with agentic traits such as being competent and assertive (Bem, 1974; Eagly, 1987; Fiske et al., 2002; Abele et al., 2008). These *competence- and warmth-related gender stereotypes* can affect evaluations of female employees and hinder them from ascending the organizational ladder (Eagly et al., 1992; Heilman, 2001, 2012). In fact, leadership represents a role that is associated with men, perceived to require agentic qualities, and to be incongruent with the female gender role (Eagly and Karau, 2002; Koenig et al., 2011). *Leader stereotypes* and the incongruence with the female gender role seem to be even more pronounced in Spain, compared to the United States or Germany (Hernandez Bark et al., 2014). A specific aspect of gender stereotypes in the work sphere that is considered in the present study is risk taking. Women are considered to lack risk propensity in general (Byrnes et al., 1999; Eckel and Grossman, 2002, 2008) and in work-related domains such as financial decision making (Beckmann and Menkhoff, 2008) or entrepreneurship (Sexton and Bowman-Upton, 1990; Verheul et al., 2012; for discussions, see Nelson, 2015; Morgenroth et al., 2017).

Stereotypes affect both other-related and self-related perceptions. On the one hand, (women) leaders may stereotype other women and men as possessing the above-indicated gender-stereotypic traits. On the other hand, women leaders themselves may feel stereotyped as women by others in their work environment. The degree to which targets of stereotypes assume that their group membership affects how people interact with them can be measured using the concept *stigma consciousness* (Pinel, 1999; Brown and Pinel, 2003). Stigma consciousness includes both the fear and the feeling that one’s behaviors in interactions are interpreted in terms of group membership.

Besides domain-specific stereotypes, in the work context there are also *negative stereotypes of mothers*. A number of studies provide evidence that women with children are evaluated more negatively as compared to men who have children or to women without children regarding their job commitment, agency, or likeability (Fuegen et al., 2004; Brescoll and Uhlmann, 2005; Heilman and Okimoto, 2008; Okimoto and Heilman, 2012). Impressions that mothers lack competence can extend from job-related abilities to parental competence and effectiveness (Heilman and Okimoto, 2008; Fuegen and Endicott, 2010; Okimoto and Heilman, 2012). Moreover, motherhood biases can result in decreased interest in hiring, promoting, or educating women who have children (Cuddy et al., 2004).

In addition to increased stereotyping, gender diversity at the workplace (or a lack thereof) can have consequences for women on a behavioral level. One example is *gender harassment* which refers to “a broad range of verbal and non-verbal behaviors not aimed at sexual cooperation but that convey insulting, hostile, and degrading attitudes about women” (Fitzgerald et al., 1995, p. 430). This includes insults, negative comments, and other negative behaviors toward women such as ignoring their contributions and interrupting them. The probability of gender harassment has been found to be higher for women whose work contexts were dominated by men, as compared to similar gender proportions (Leskinen et al., 2011; Kabat-Farr and Cortina, 2014).

To gain a broader impression of the effects of gender proportions, a consideration of women’s *work-related well-being* and overall quality of life is important. Several studies have focused on how job satisfaction, a common operationalization of work-related well-being (Warr, 2013), is influenced by gender diversity at the workplace. Yet, no clear answer has been found for whether gender diversity has primarily positive or negative effects for employees’ job satisfaction (for reviews, see Williams and O’Reilly, 1998; Tolbert et al., 1999; Peccei and Lee, 2005). It has been suggested that the relation between gender diversity and job satisfaction is moderated by the organizational climate (Miner-Rubino et al., 2009). In negative climates, an increasing number of women on higher organizational levels went along with less job satisfaction for women. In contrast, women’s job satisfaction increased with more women on higher organizational levels in positive climates. In a more recent study, lower percentages of men in the occupation were related to higher levels of affective well-being for women (Qian and Fan, 2018).

A possible consequence of a negative organizational climate could be *work–family conflict*. Past research has shown that women’s well-being and job satisfaction are affected by work–family conflict (Kossek and Ozeki, 1998; Allen et al., 2000; Grant-Vallone and Donaldson, 2001; Bruck et al., 2002; Ford et al., 2007; Shockley and Singla, 2011). For example, less work–family conflict or even enrichment resulting from the availability of flexible work arrangements can increase employees’ job satisfaction (Carlson et al., 2010; McNall et al., 2010). This is especially true for women (Carlson et al., 2010) and for work interference with family in contrast to family interference with work (Allen et al., 2013). Moreover, gender composition affects work–family conflict as token women experience increased work–family spillover (Maume and Houston, 2001).

When employees experience work–family conflict, emotional responses such as increased guilt are often the consequence (Judge et al., 2006; Livingston and Judge, 2008). Again, pressure to be a perfect parent and *feelings of guilt* are particularly common for women and mothers (Sutherland, 2010; Borelli et al., 2017; Meeussen and Van Laar, 2018). In the absence of gender diversity, these feelings could be augmented due to the accompanying adherence to stereotypes and traditional role expectations for women.

## The Present Study

The aim of the present study was to test the perception of a range of negative consequences depending on the proportion of women and men on the top leadership level in organizations. Our convenience sample consisted of women in leadership positions in Spain from different companies and organizations. They reported whether there was a male majority, a female majority, or similar proportions of both genders at the top level in their organization (as well as on their own leadership level). Special efforts were taken to recruit women from organizations that are as of yet under-researched: those with female majorities at the top level.

On the basis of previous tokenism studies, we included the nine outcome variables described below. First, we assessed traditional gender stereotypes regarding (1) warmth and (2) competence, (3) stigma consciousness, and (4) gender harassment. In addition to these general dimensions of traditional gender stereotypes, because leadership abilities (Schein, 2001; Heilman and Okimoto, 2007; Eagly and Sczesny, 2009), including risk propensity (Eckel and Grossman, 2002, 2008), are specific positive aspects of traditional male stereotypes that are relevant at work, we also assessed (5) women-leader stereotypes. Because (6) negative stereotypes of mothers (e.g., Cuddy et al., 2004) appear widespread in work-contexts, these were also measured. In addition, we measured (7) work-related well-being and (8) work–family conflict, along with the (9) perception that other women feel guilty regarding family obligations (e.g., Borelli et al., 2017). We define as negative outcomes: higher stigma consciousness, more gender harassment, more negative stereotypes of women as leaders, more negative stereotypes of mothers, lower work-related well-being, higher work–family conflict, and other women’s higher perceived guilt. It is debatable whether more stereotyping in general is a negative outcome, or whether positive stereotypes of women’s competence and warmth are positive outcomes (see section “Discussion”).

Based on the theorizing delineated above, we expected more positive outcomes in the presence of gender diversity (i.e., similar proportions of both genders) than in the presence of a male or a female majority. In other words, as the reported proportion of women at the top level of the organization increases (from skewed via tilted to balanced), reported outcomes should become more positive; but then again outcomes should become more negative as men become the minority. Statistically, this U-shaped function should manifest in analyses of variance (i.e., planned contrasts) in quadratic trends of gender diversity (measured with five levels, see below). Alternatively, it is possible that outcomes are perceived more positively the higher the proportion of women (i.e., of ingroup members). This alternative hypothesis would statistically manifest in linear trends instead.

## Materials and Methods

### Participants

Target participants were women working in leadership positions in medium-sized or large companies or organizations in Spain (i.e., with more than 50 employees). They were contacted online and invited to take part in an online “study on gender and leadership at work.” To reach a broad and diverse sample, ways of recruitment were: via a women’s association (Associació Dones en xarxa/Mujeres en red, 149 respondents); via a top-level professional woman’s personal networks (65 women); and via a professional data collection company (Opinòmetre; 511 respondents finished, 136 dropped out: response rate among the latter: 79%). Women indicating to occupy low-level positions were then excluded from data analyses, and so were those from organizations with fewer than 50 employees.

The mean age of the final sample of *N* = 649 was 39 (*SD* = 9.8, range: 18–71 years). Among them, 160 women (25%) indicated to occupy high-level positions in the workplace, and 489 women (75%) indicated to hold medium-level positions. Because of the different organizations and sectors targeted, we distinguished only these two leadership levels that we reasoned should exist in any organization medium-sized or larger, and the participants themselves chose the adequate level. A further description of the sample is presented in Table 1. We computed a dichotomous variable, low (51%) versus high family obligations (49%). We defined high family obligations as elderly care, at least one child under 11 years of age (reported by 38%), or both.

**Table 1:** Demographic characteristics of the sample of women in leadership positions.

Demographic characteristic	Whole sample (*N* = 649)	Top level (*n* = 160)	Intermediate level (*n* = 489)
Age (years)	39	40	39
University degree	90%	96%	88%
Field of study:			
Gender-typical	66%	68%	67%
Not gender-associated	14%	11%	14%
Counter-stereotypical	20%	21%	19%
Sector:			
Technical, education, science	33%	26%	35%
Health, social	27%	26%	27%
Industry, construction	21%	20%	21%
Finance, insurances, marketing	16%	24%	14%
Other	3%	4%	3%
Private situation:			
Have a partner	84%	91%	82%
Have children	75%	85%	72%
Care for an elderly family member	20%	26%	18%

*Because of missing data, some ns are smaller.*

On a 1–7 scale, the proportions of women/men working at the highest level were reported as: only men (i.e., 1): 5%, 2: 23%, 3: 31%, 4, equal proportions: 13%, 5: 19%, 6: 8%, 7, only women: 2%. Because of the small percentages responding at the extremes, we formed five groups, summing responses 1–2 and 6–7. As a side note, the proportions of women/men reported at the intermediate level were similar, and the correlation between both proportions was high (*r* = 0.56 for participants at the intermediate level), indicating that as a rule, a high proportion of women at the top went along with a high proportion at the intermediate level, too. All patterns of findings reported below are similar if gender diversity at one’s own level is considered.

### Ethical Considerations

This study was carried out in accordance with the recommendations of the German Psychological Society (Deutsche Gesellschaft für Psychologie, DGPs) according to which ethics approval was not required for this study. Specifically, our Ethics Committee at the Faculty of Psychology, University of Koblenz-Landau, undertakes a full examination of a research study only if at least one of 18 conditions of potential ethical concern is met (most importantly, underage participants, deception involved, psychological strain, no full debriefing, no informed consent, health risks, no confidential treatment of data). None of these conditions of potential ethical concern were met in the present study, so no full examination by the Ethics Committee was asked for. Online informed consent was obtained from all participants. All subjects were treated in accordance with the Declaration of Helsinki and informed in advance that we were only interested in their personal opinions, that data would only be subjected to group-based analyses, and that their data would be treated confidentially; they were free to drop out by leaving the online questionnaire any time.

### Procedure and Materials

Once informed consent was obtained, the questionnaire started with socio-demographic questions (see section “Participants”). Then, several scales were administered in the order described below, all using Likert-type scales anchored with 1 = “do not agree at all” and 7 = “completely agree,” unless mentioned otherwise. Several additional scales were included that are irrelevant to the present paper and are therefore not described here. The data are available, omitting the demographic information^1^.

#### Work-Related Well-Being

Well-being, stressing work-related factors (satisfaction with work, financial situation, relationships with co-workers, opportunities for promotion), was measured averaging four items (e.g., translated from Spanish as “indicate how satisfied you are with your financial situation,” anchored 1 = “not satisfied at all,” 7 = “completely satisfied,” Cronbach’s α = 0.77); items were taken from the Life satisfaction questionnaire (Fahrenberg et al., 2000). A fifth item related to well-being, “indicate how satisfied you are with your health” correlated highly with the scale mean, *r* = 0.41, but was excluded from the scale to arrive at a conceptually cleaner construct.

#### Traditional Gender Stereotypes

Traditional gender stereotypes associating men with competence were measured averaging the three items competent, efficient, and able (e.g., “indicate the degree to which you think men and women are competent,” anchored 1 = “applies more to men,” 7 = “applies more to women,” Cronbach’s α = 0.83). Traditional gender stereotypes associating women with warmth were measured using seven items (e.g., warm, trustworthy, α = 0.86, both after Runge et al., 1981).

#### Gender Harassment

Three items were used to measure the perceived prevalence of gender harassment against women in the company or organization (Cronbach’s α = 0.91), based on Yoder (2001, 2002), for example, “Compared to men, at meetings (or similar events) women are interrupted more often.”

#### Negative Women-Leader Stereotypes

We used seven items (Cronbach’s α = 0.89) to measure participants’ own negative work and leadership-related stereotypes of women, four from the Social Role Questionnaire (Baber and Tucker, 2006), for example, “Some types of work are just not appropriate for women.” Three additional items regarding risk taking (Eckel and Grossman, 2002, 2008) were formulated for the present study, for example, “Male leaders are better able than female leaders to make risky decisions.” Both aspects, leadership and risk-taking, correlated highly (*r* = 0.65), the internal consistency of the whole scale was higher than that of each aspect, and findings were comparable if separate scales were formed; therefore, we report findings of the composite scale.

#### Negative Stereotypes of Mothers

Others’ perceived negative work-related stereotypes of mothers were measured with three items (Cronbach’s α = 0.92, modeled after Fuegen and Endicott, 2010), for example, “If a woman has children, others think she will not work enough hours.”

#### Stigma Consciousness

Six items were used to measure in how far a woman perceives that she is stereotyped and discriminated at work (Cronbach’s α = 0.85), for example, “Some of my colleagues feel that I have less ability because I’m a woman” (Von Hippel et al., 2011).

#### Work–Family Conflict

Two items were used to measure how far women strived for more work flexibility (“I would like more flexibility in my job that would allow me to satisfy the needs of my family,” “I would like a job that allows me to choose working hours more flexibly,” Cronbach’s α = 0.89), that we formulated based on a European Union Survey (2010).

#### Other Women’s Perceived Feelings of Guilt

We finally asked, “If women, because of their work schedule, do not manage reconciling work and family needs, they feel bad and guilty (in other words, if they cannot take part in activities with the children, cannot take care of them or accompany them).” This item was based on works by Rodríguez et al. (2009) and Berlanga et al. (2013).

## Results

In all analyses in the present article, significance tests were conducted with α ≤ 0.05. As an indicator of the effect size, *R*^2^_p_ is reported (see Cohen, 1977). Effects with sizes under *R*^2^_p_ = 0.02 are not discussed because of little practical significance. A preliminary MANOVA indicated multivariate effects of age and own family obligations, and interactions of own level with proportion of women at the top. Therefore, age and own family obligations were controlled in all analyses, and level was treated as an additional independent variable. Thus, we computed 2 × 5 ANOVAs with own level (intermediate vs. top) and proportion of women at the top (five levels: hardly any to almost all) treated as IVs, controlling for age and family obligations (dichotomized: in charge of children under 11 years and/or old people, or not). Polynomial contrasts were used to test the linear and quadratic trends.

### Linear Trends: Quality of Life and Stereotypes

**FIGURE 1:**
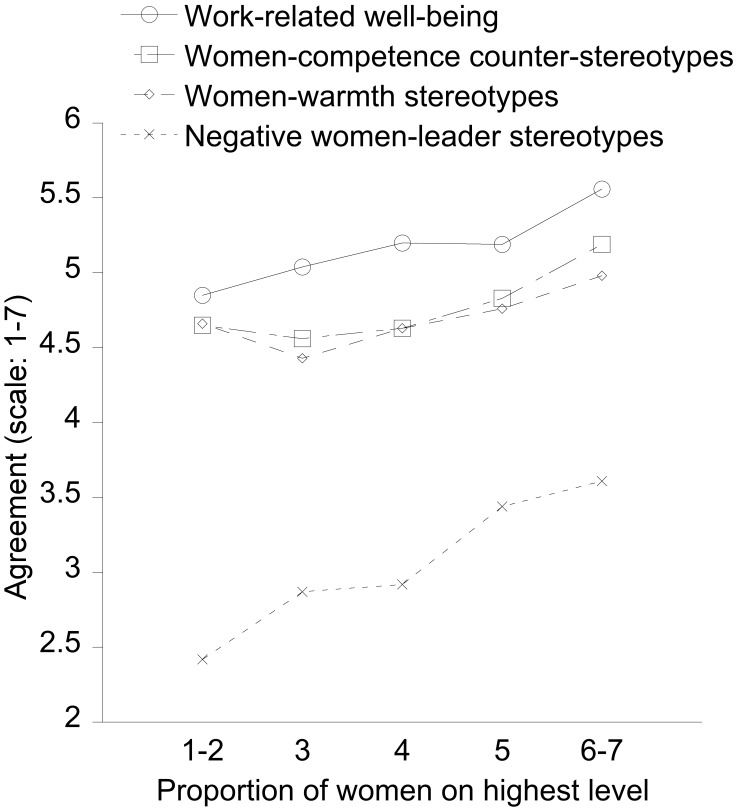
Degree to which women in leadership positions (both on the highest and intermediate level), depending on the proportion of female leaders on the highest level in their organization (scale: 1–2 – almost only men, to 6–7 – almost only women), reported high work-related well-being, and endorsed positive women-competence counterstereotypes and women-warmth stereotypes, as well as negative stereotypes of women as leaders. All variables show linear trends, with increasing agreement (1 = “do not agree at all” to 7 = “completely agree”) going along with increasing proportions of women leaders.

We first present the outcome variables for which our main hypothesis was not supported. As Figure 1 shows, several positive effects appear to be linearly related to the proportion of women at the top level of the organization.

#### Work-Related Well-Being

The ANOVA showed that work-related quality of life (DV) was higher the higher the proportion of female leaders; main effect of proportion of women at the top: *F*(4,635) = 4.70, *p* = 0.001, *R*^2^_p_ = 0.03, linear trend: estimate: 0.49, *SE* = 0.12, *p* < 0.001. The only other statistically significant effect was that women in top positions reported higher quality of (work) life than those in intermediate positions; main effect of own level, estimated marginal means: *M*s = 5.36 and 4.98 (*SE*s = 0.08 and 0.05), *F*(1,635) = 14.79, *p* < 0.001, *R*^2^_p_ = 0.02. A supplementary analysis using the single-item “health-related well-being” (see “Materials and Methods”) did not replicate this main effect (*F* = 1.60, *p* = 0.17).

#### Traditional Gender Stereotypes of Competence and Warmth

The same ANOVA as above, but with repeated measures on the DV general gender stereotypes (women-competence counter-stereotypes versus women-warmth stereotypes), also showed a main effect of proportion of women at the top level, *F*(4,635) = 7.57, *p* < 0.001, *R*^2^_p_ = 0.05, linear trend: estimate: 0.37, *SE* = 0.08, *p* < 0.001. As Figure 1 shows, the higher the proportion of women in the organization, the more women-competence counter-stereotypes and the more women-warmth stereotypes women leaders reported. An additional quadratic trend (estimate: 0.25, *SE* = 0.08, *p* = 0.002) indicated that there was more stereotyping (both regarding competence and warmth) with hardly any women (scores 1–2) at the top than with a minority of women (3, see left-most points in Figure 1). In addition, women in top positions reported somewhat more stereotypes altogether than those in intermediate level positions, main effect of own level: *M*s = 4.81 and 4.65, *SE*s = 0.06 and 0.04, *F*(1,635) = 4.89, *p* < 0.03, *R*^2^_p_ = 0.01; and women with high family obligations (covariate) reported somewhat more stereotypes than those with low family obligations, *F*(1,635) = 6.14, *p* = 0.01, *R*^2^_p_ = 0.01. Both of the latter effects explain very little variance and will thus not be interpreted. There were no other effects, implying that none of the effects interacted with competence/warmth stereotypes (all *F*s < 2).

#### Negative Women-Leader Stereotypes

In line with this impression of more stereotyping in general, both regarding women-competence counter-stereotypes and traditional women-warmth stereotypes, with increasing proportions of women at the top of the organization, negative stereotypes of women as leaders increased as well, *F*(4,635) = 9.60, *p* < 0.001, *R*^2^_p_ = 0.06, linear trend: estimate: 0.93, *SE* = 0.16, *p* < 0.001). Agreement with negative stereotypes was generally low, however. In addition, both covariates were statistically significant: Older women reported somewhat fewer negative female-leader stereotypes than younger women did, *F*(1,635) = 3.89, *p* < 0.05, *R*^2^_p_ = 0.01, and women with family obligations reported more negative women-leader stereotypes than those without family obligations, *F*(1,635) = 9.96, *p* = 0.002, *R*^2^_p_ = 0.02.

### Quadratic Trends: Positive Effects of Gender Diversity

**FIGURE 2:**
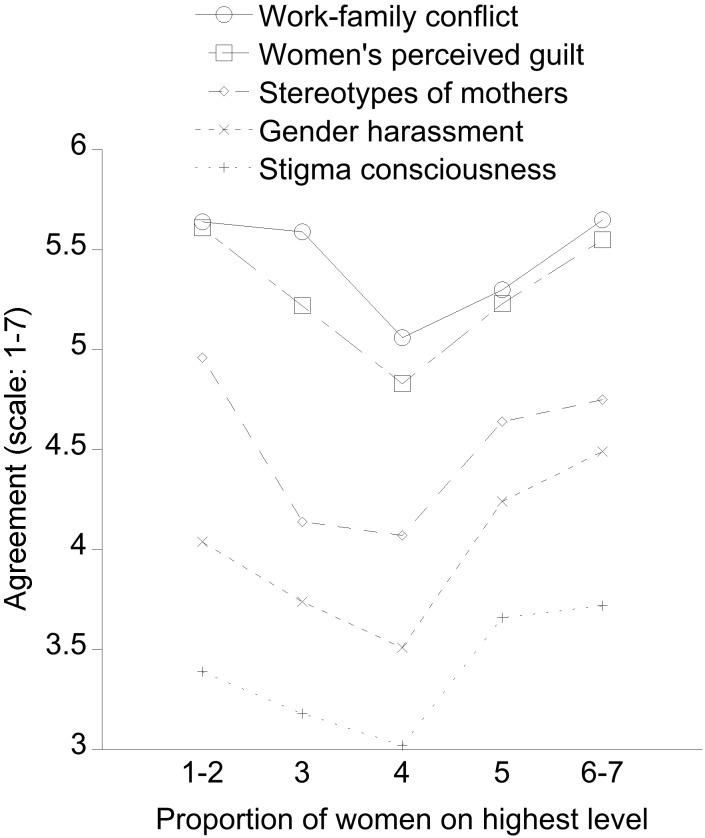
Degree to which women in leadership positions (both on the highest and intermediate level), depending on the proportion of female leaders on the highest level in their organization (scale: 1–2 – almost only men, to 6–7 – almost only women), feel work–family conflict, perceive that other women feel guilty because of their work–family obligations, report that negative stereotypes of mothers and gender harassment exist in their organization, and report to be stigma conscious. All variables show quadratic trends, with lowest agreement (1 = “do not agree at all” to 7 = “completely agree”) going along with similar proportions of men and women leaders (i.e., gender equality).

As Figure 2 shows, in line with our hypothesis, several outcomes that indicate negative perceptions were highest with lowest and highest proportions of women, and lowest with gender diversity (i.e., equal proportions of women and men).

#### Work–Family Conflict

The ANOVA on women’s own work–family conflict showed a main effect of proportion of women at the top level, *F*(4,635) = 3.13, *p* < 0.02, *R*^2^_p_ = 0.02, that was due to a quadratic trend (estimate: 0.42, *SE* = 0.15, *p* < 0.01). Women reported the least work–family conflict if similar proportions of female and male leaders worked at the top of their organization. In addition, there was a statistically but not practically significant main effect of the covariate age, *F*(1,635) = 5.48, *p* = 0.02, *R*^2^_p_ < 0.01, indicating that work–family conflict was lower for women of higher age.

#### Other Women’s Perceived Feelings of Guilt

The same ANOVA on other women’s perceived feelings of guilt showed a main effect of proportion of women at the top level, *F*(4,608) = 3.09, *p* < 0.02, *R*^2^_p_ = 0.02, that was again due to a quadratic trend (estimate: 0.59, *SE* = 0.18, *p* = 0.001). Women perceived that other women felt less guilty about not managing to reconcile work and family needs if similar proportions of female and male leaders worked at the top of their organization. In addition, there was a small main effect of the covariate own family obligations of little practical significance, *F*(1,608) = 4.66, *p* = 0.03, *R*^2^_p_ < 0.01, indicating that women with higher family obligations tended to perceive higher guilt than those with lower family obligations.

#### Negative Stereotypes of Mothers

The same ANOVA on stereotypes of mothers showed a main effect of proportion of women at the top level, *F*(4,635) = 5.49, *p* < 0.001, *R*^2^_p_ = 0.03, that was also due to a quadratic trend (estimate: 0.67, *SE* = 0.19, *p* = 0.001). Women held the least negative stereotypes of mothers if similar proportions of female and male leaders worked at the top of their organization. In addition, a small main effect of the covariate own family obligations, *F*(1,635) = 5.32, *p* = 0.02, *R*^2^_p_ < 0.01, showed that women with higher family obligations perceived negative stereotypes of mothers to be higher than women with lower family obligations did.

#### Gender Harassment

The ANOVA on gender harassment also showed a main effect of proportion of women, *F*(4,635) = 4.43, *p* = 0.002, *R*^2^_p_ = 0.03, again due to a quadratic trend (estimate: 0.55, *SE* = 0.18, *p* = 0.003), in addition to a linear trend (estimate: 0.44, *SE* = 0.18, *p* < 0.02). Women reported the least gender harassment in their organization if similar proportions of female and male leaders worked at its top, but more gender harassment was perceived with a female majority at the top level as compared to a male majority. We also found a small main effect of own leadership level, *F*(1,635) = 7.90, *p* < 0.01, *R*^2^_p_ = 0.01, as well as an interaction of leadership level with proportion of women at the top level, *F*(4,635) = 2.45, *p* < 0.05, *R*^2^_p_ = 0.02. Women at the top level reported more gender harassment than women at the intermediate level did (*M*s = 4.22 vs. 3.78). Details of the unexpected interaction are reported in the Appendix. In addition, there was a small main effect of the covariate own family obligations, *F*(1,635) = 4.99, *p* < 0.03, *R*^2^_p_ < 0.01: Women with higher family obligations perceived more gender harassment than women with lower family obligations did.

#### Stigma Consciousness

Finally, the ANOVA on own stigma consciousness showed a main effect of proportion of women, *F*(4,635) = 2.97, *p* < 0.02, *R*^2^_p_ = 0.02, that was due to a quadratic trend (estimate: 0.55, *SE* = 0.18, *p* = 0.003), in addition to a linear trend (estimate: 0.36, *SE* = 0.18, *p* < 0.05). Women reported the least stigma consciousness if similar proportions of female and male leaders worked at the top of their organization, but more stigma consciousness was perceived with a female majority as compared to a male majority. In addition, there was a main effect of the covariate own family obligations, *F*(1,635) = 12.34, *p* < 0.001, *R*^2^_p_ = 0.02, showing that women with higher family obligations had higher stigma consciousness than women with lower family obligations; and a small main effect of the covariate age, *F*(1,635) = 4.75, *p* = 0.03, *R*^2^_p_ < 0.01, indicated that older women reported lower stigma consciousness than younger women.

## Discussion

Based on a heterogeneous sample of women in leadership positions in Spain, the present study tested Kanter’s (1977) seminal gender-diversity hypothesis on various outcome variables: In spite of all other possible differences between companies and organizations (e.g., sizes, business sectors), can we find evidence for the hypothesis that outcomes are more positive in the presence of gender diversity than with a male or female majority? Gender diversity was operationalized as similar proportions of both genders at the top leadership level of the organization (reported by participants), and outcomes were related to gender stereotyping, gender roles, and work-related well-being. Some outcomes were women’s own endorsements (e.g., their personal gender stereotypes), others were their perceptions of the climate in their organization (e.g., regarding stereotypes of mothers).

With regard to eight of the nine hypotheses tested, we found some evidence for Kanter’s (1977) hypothesis. We first consider the presence of gender diversity at the top level of the organization, as compared to a male majority. Women reported higher work-related well-being as well as more positive own stereotypes of women’s, as compared to men’s, competence and warmth. They also reported to experience lower levels of work–family conflict and perceived other women to feel less guilty about neglecting their families if they had work obligations; they reported to perceive the lowest levels of negative work-related stereotypes of mothers, to perceive the lowest levels of gender harassment, and they were the least stigma conscious; in other words, they did not feel others stereotyped them as women in work-related interactions.

We had assumed that not only a male majority, but also a female majority at work has negative consequences, because gender becomes salient in both cases. However, at the other end of the tail, comparing gender diversity with female majorities at the top level, findings were more mixed. Specifically, higher percentages of women leaders went along with higher work-related well-being as well as more positive own stereotypes of women’s, as compared to men’s, competence and warmth. In contrast, work–family conflict, perceived guilt, negative work-related stereotypes of mothers, gender harassment, and stigma consciousness were reported to be more positive with gender equality than with a female majority. The final hypothesis was not corroborated in any theoretically expected way: Own negative stereotypes of women as leaders, including risk propensity, were higher the higher the proportion of female leaders. In the following, we discuss each of these findings.

For all outcome variables that were related to the perceived organizational climate, broadly defined, the hypothesis that gender diversity is related to the most positive climate was supported. Specifically, the outcomes were the perception that, in one’s organization, gender harassment is frequent, that others hold negative stereotypes of mothers, and the perception that other women feel guilty because of work–family conflict. Two additional outcomes supported the gender diversity hypothesis: own work–family conflict and own stigma consciousness. Taken together, these outcomes provide encouraging evidence for the gender diversity hypothesis, showing that gender diversity at work is related to manifold positive consequences. In line with Kanter’s (1977) theorizing, gender appears least visible with similar numbers of women and men, gender differences seem least exaggerated, and there seems to be the least stereotyping regarding family-related gender roles.

What distinguishes these outcomes supporting the gender diversity hypothesis from those that did not support the hypothesis? The first set of findings that did not support the gender diversity hypothesis can be summarized as own stereotypes. Importantly, we phrased those questions generally and did not ask about stereotypes in one’s organization (e.g., “in your work environment, indicate the degree to which you think men and women are competent”). Stereotypes are notoriously slow to change (e.g., Kunda and Oleson, 1995), which could explain why gender diversity in the organization is not related to little stereotyping among the women working there. But why did we observe linear trends, with higher stereotypes reported in the presence of higher proportions of female leaders? We speculate that this could be due to other, uncontrolled differences between organizations, for example, the business sector. As a side note, competence and warmth stereotypes were higher with token proportions of women (see Figure 1, 1–2) than with a higher proportion, supporting previous findings (Hewstone et al., 2006, 2011). As another side note, in contrast to our other outcome variables, higher positive stereotypes of women cannot clearly be evaluated as positive or negative outcomes because stereotyping, even if positively toned, may have negative consequences (e.g., Pratto et al., 1997; Jost and Kay, 2005).

Regarding stereotypes, a particularly puzzling finding is that both positive women-competence counterstereotypes and negative women-leader stereotypes were higher the higher the proportion of women leaders. A possible explanation could be the generality versus specificity of the stereotype at hand. General competence-stereotypes have been reported to change with women’s changing gender roles, whereas more specific stereotypes change more slowly, if at all (e.g., Kessels et al., 2006; Steffens and Jelenec, 2011; Ebert et al., 2014). For example, the specific stereotypes in previous research pertained to STEM fields (i.e., science, technology, engineering, and math). Applied to the present findings, higher proportions of women leaders would go along with changes in general impressions of women’s competence, but not with more specific changes regarding their leadership ability. As far as leadership abilities are concerned, we also think that risk propensity (that we included in the scale) is often discussed in a normative way, as if risky decision making were generally good and risk aversion, generally bad (for discussion, see Steffens and Viladot, 2015). However, if the women in our sample agreed that male leaders have a higher risk propensity than female leaders, it is an open question whether they thought this was a good thing. Alternatively, the presence of more negative gender stereotypes with higher proportions of female leaders could also second the conclusions of a previous study that the mere presence of more women in key senior positions is insufficient to change inequality if bias in the organization is not tackled (Sterk et al., 2018).

In the presence of gender diversity, several aspects of the perceived organizational climate were better, and women reported less work–family conflict and less stigma consciousness at work, which could suggest higher work-related well-being, too. However, a linear instead of a U-shaped trend was found for work-related well-being. In order to understand this pattern, we supplemented the analysis above with one where we used only one item, “how satisfied are you with your work?” instead of the whole scale. The pattern was identical to the linear trend reported. Again, other confounding variables, such as the business sector, could be responsible for that trend that we cannot explain. Another speculation is that in jobs in which stereotype threat is “in the air,” such as leadership positions, the proportion of women surrounding a female leader is reassuring by itself (for related evidence, see Inzlicht and Ben-Zeev, 2000; Peccei and Lee, 2005; London et al., 2012). This would explain why the women in our sample reported higher work-related well-being the higher the proportion of female leaders in their organization.

The unexpected finding that more gender harassment goes along with particularly high proportions of women at the top can be interpreted as evidence for the threat hypothesis (Yoder, 1991). The women in our sample reported the highest incidence of gender harassment in the presence of female majorities, and this was particularly the case for women working at the top level (see Appendix). Possibly, men feel threatened by being in the minority in positions that were traditionally “their turf” (i.e., leadership), and consequently, there is more gender harassment than the mere numbers would suggest (for basic research on the relationship between men’s threat and gender harassment, see e.g., Maass et al., 2003).

We used own family obligations and age as covariates in all analyses because these demographic characteristics showed statistically significant effects on some of the variables we considered as outcomes. Age effects will not be discussed because in no instance did the covariate explain a practically significant proportion of the variance. Many of the effects of own family obligations were negligible, too, but women with family obligations reported more negative stereotypes of women leaders and more stigma consciousness. In other words, their perceptions of the work environment were, if anything, more negative than perceptions of women with fewer obligations besides work. A possible reason for this finding is that women with family obligations are often reminded that they do not represent “the ideal worker” who can always make work a top-priority, which might remind them of their gender and of the limitations related to traditional female gender roles (also see Ellemers et al., 2012).

Some limitations of the present study need to be mentioned. First, the effects we found were generally small in terms of the amount of variance explained. We believe that this is to be expected due to the many uncontrolled differences present in a broad and diverse sample as the present one – for example, different organizations of different sizes in different sectors. In light of a host of uncontrolled influences, we believe the effects shown in Figure 2 are quite substantial, with gender diversity often leading to an average improvement of nearly one unit (on a seven-point scale) or effect sizes of up to Cohen’s *d* = 0.33. Still, a replication in which many of these influences are controlled would be a valuable addition, for example, in an international company that shares many features, but where the top national level consists of mainly men, mainly women, or is gender diverse. Such a study would also overcome the present limitation that we collected data only in one culture, Spain. In such a replication, the coarse operationalization of “leadership level” that we used could also be improved. Because of the many differences that may exist between organizations, we only distinguished the intermediate from the top leadership level and collected subjective estimates. Better estimates can be obtained from employees if the number of levels present in their organization is adequately represented by the questionnaire. Nevertheless, note that the most likely consequence of a better operationalization would be stronger effects than those reported here.

Another important limitation is that all our findings are cross-sectional and thus merely correlational; only a longitudinal study – or, if possible, an experiment – could determine whether gender equality is actually the cause of the positive outcomes that we report for organizations with equal proportions of female and male leaders. It is also a limitation that the proportions of women and men present at the top and intermediate leadership level were highly correlated. It is thus unclear whether gender diversity at the top level or at one’s own level is related to the outcomes we reported. Moreover, the present study completely relied on self-report, lacking any objective outcome measures. Finally, whereas we administered some scales validated by previous research, others were developed for the present purposes, with questionable validity. Therefore, the findings deserve independent replication.

A theoretical implication of the present findings is that they strengthen the empirical basis of Kanter’s (1977) hypothesis: Equal proportions of women and men leaders in organizations go along with more positive perceptions pertaining to the organizational climate than male majorities do. A practical implication is that the findings provide additional evidence on the “value in diversity” hypothesis (e.g., Ehrke et al., 2014). Obtaining equal chances for both genders is not only a matter of fairness, but gender diversity is also related to more positive outcomes for female leaders than other proportions (i.e., either male or female majorities). Our findings thus support establishing gender quotas or other measures to increase diversity that are often discussed controversially. The findings stress the importance for organizations to include diversity in their organizational values and implement instruments of diversity management.

To conclude, we used a variety of outcome variables and found, regarding most of them, support for Kanter’s (1977) theorizing that gender equality at the top of organizations goes along with more positive perceptions among its female leaders than a male majority does. Regarding female majorities at the top, the evidence was more mixed: The organizational climate was perceived more positively in various ways in the presence of gender diversity, but this did not extend to women’s endorsement of gender stereotypes and their work-related well-being. We hope that future research establishes and clarifies these connections.

## Ethics Statement

This study was carried out in accordance with the recommendations of the Ethics Committee, Faculty of Psychology, University of Koblenz and Landau. Informed consent was obtained from all subjects prior to clicking the link to the study. All subjects were treated in accordance with the Declaration of Helsinki and informed in advance that we were only interested in their personal opinions, that data would only be subjected to group-based analyses, and that their data would be treated confidentially.

## Author Contributions

MV and MS planned the study and designed the questionnaire. MV collected the data. MS analyzed the data. MS and CS drafted the manuscript. MV, CS, and MS revised and approved the manuscript.

## Conflict of Interest Statement

The authors declare that the research was conducted in the absence of any commercial or financial relationships that could be construed as a potential conflict of interest.

## Figures and Tables

**FIGURE A1:**
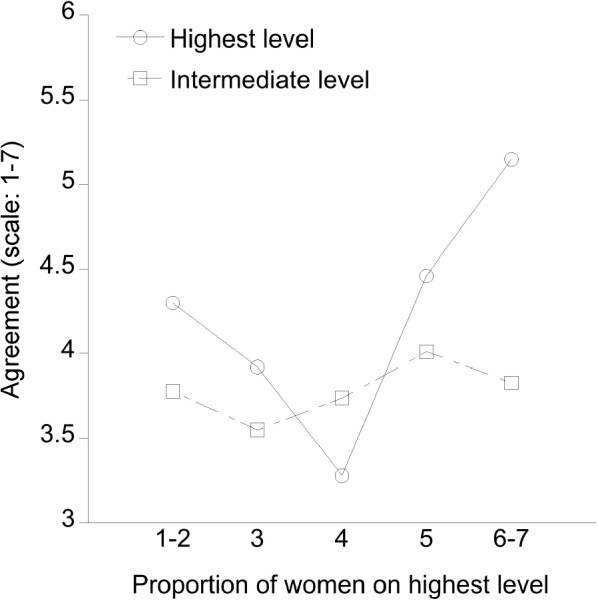
Visualization of the interaction between proportion of women at highest level in one’s organization (scale: 1–2 – almost only men, to 6–7 – almost only women) and own level (highest vs. intermediate level) on reported gender harassment. Findings show that women on the highest level report most gender harassment in their organization (agreement: 1 = “do not agree at all” to 7 = “completely agree”) in the presence of a majority of women leaders.

## Footnotes

1 https://osf.io/gwt3s/

## Appendix

Figure A1 depicts the unexpected interaction. As can be seen, the quadratic trend is clearer for women at the highest leadership level. Those at the intermediate level report less gender harassment in the presence of very high numbers of women (i.e., 6 or 7) than in the presence of high numbers (i.e., 5). Women at the highest level of leadership report more gender harassment than those at the intermediate level except with equal proportions of women and men. Tests of simple main effects indicated that proportion of women had a statistically significant effect only for the gender harassment reported by women on the highest level, *F*(1,635) = 4.37, *p* < 0.01, *R*^2^_p_ = 0.03. And the difference between levels was statistically significant only with the highest proportion of women, *F*(1,635) = 8.80, *p* < 0.01, *R*^2^_p_ = 0.01 (other *F*s < 2.71, *p*s > 0.10). We believe these unexpected interactions should be replicated before interpretation.
